# Levofloxacin Versus Ciprofloxacin in the Treatment of Urinary Tract Infections: Evidence-Based Analysis

**DOI:** 10.3389/fphar.2021.658095

**Published:** 2021-04-08

**Authors:** Dehong Cao, Yinzhi Shen, Yin Huang, Bo Chen, Zeyu Chen, Jianzhong Ai, Liangren Liu, Lu Yang, Qiang Wei

**Affiliations:** ^1^ Department of Urology, Institute of Urology, West China Hospital, Sichuan University, Chengdu, China; ^2^ West China School of Medicine, Sichuan University, Chengdu, China

**Keywords:** urinary tract infection, fluoroquinolone, levofloxacin, ciprofloxacin, efficacy, safety

## Abstract

Urinary tract infections (UTIs) are one of the most common bacterial infections acquired both in community and hospital. Fluoroquinolones, represented by levofloxacin and ciprofloxacin, are widely used for treatment of UTIs. However, it remains controversial for the comparison between the 2 drugs, which propelled us to conduct the first evidence-based research on this topic. To establish their relative efficacy and safety, we searched Pubmed, embase, and Web of Science for randomized controlled trials (RCTs) for UTIs. A total of 5 RCTs were finally included, involving 2,352 patients and a systematic review and meta-analysis were performed to compare the end-of-therapy and posttherapy clinical success rate, microbial eradication rate and adverse event rate. Jadad score and Review Manager 5.3.0 version were applied respectively to evaluate the study quality and heterogeneity. There was no significant difference between levofloxacin and ciprofloxacin group in end-of-therapy or posttherapy clinical success rate and microbial eradication rate (*p* > 0.05). As for adverse event rate, the 2 drugs were comparable and both safe for clinical use. Based on one included trial and pharmacological research, we raised hypothesis that levofloxacin was superior to ciprofloxacin for treatment of E. coli-induced chronic bacterial prostatitis (CBP) and it required a further study to prove it.

## Introduction

UTIs consists of complicated urinary tract infections (cUTIs) and the uncomplicated urinary tract infections (including prostatitis, pyelonephritis and cystitis). And cUTIs are often associated with abnormal urogenital system structure or function (Foxman, 2010). UTIs are some of the most common bacterial infections, affecting 150 million people each year worldwide. In the United States, the societal costs of these infections, including health care costs and time missed from work, are approximately US$3.5 billion per year (Flores-Mireles et al., 2015). Women are much more sensitive to UTIs (Stamm and Norrby, 2001) and studies have reported that 40∼50% of women worldwide will suffer from UTIs at least once in their lifetime. UTIs are identically harmful for men, especially reproductive function. CBP, a specific type of UTIs, has negative effect on sperm motility and morphology (Rusz et al., 2012). Compared with normal ejaculate, a higher leukocyte count could be observed in CBP patients (Schuppe et al., 2017), which is associated with the pathophysiological changes of sperm damage (La Vignera et al., 2014). Once spread to the accessory gland, UTIs could cause a great decline of total sperm number and bilateral infection is more detrimental (Vicari et al., 2006; La Vignera et al., 2011).

Levofloxacin and ciprofloxacin are antimicrobial agents and are expected to develop a widened use for its underlying effect in neuroinflammation modulating (Zusso et al., 2019), hematopoietic stem cell transplantation (Rambaran and Seifert, 2019), and even inhibition of SARS-CoV-2 replication (Karampela and Dalamaga, 2020). With respect to UTIs, the therapeutic effect of fluoroquinolones has been proved by many studies (Bader et al., 2017; Chu and Lowder, 2018; Bientinesi et al., 2020). Studies show that in Asian countries, 24.1% of patients with UTIs were given fluoroquinolones, second only to cephalosporin antibiotics (34.4%) (Choe et al., 2018a). The guidelines of the Urological Association of Asia list fluoroquinolones as the first choice drug for pyelonephritis (LE:1A,GR:A) (Choe et al., 2018b). Among fluoroquinolones, levofloxacin and ciprofloxacin are most commonly used in the treatment of acute pyelonephritis (AP) and cUTIs (Kranz et al., 2018).

Levofloxacin and ciprofloxacin are both recommended for clinical application in UTIs and, though commonly prescribed, there’s no final conclusion on the comparative merit of the either one. Levofloxacin shows advantage over ciprofloxacin in terms of efficacy, disease reoccurrence and adverse event (Zhang et al., 2012). On the contrary, microbiology evidence shows that the uropathogen is more sensitive to ciprofloxacin (Afriyie et al., 2018; Humphries et al., 2019). Currently, no evidence-based medical research has been published worldwide on this topic, which makes our study the first systematic review and meta-analysis in the world. Our objective was to compare the efficacy and safety of the two drugs in the treatment of UTIs, by performing a meta-analysis of high-quality RCTs that compared levofloxacin and ciprofloxacin.

## Materials and Methods

### Search Strategy

We performed a systematic literature search using the PubMed, Web of Science and embase databases up to January, 2021. We restricted our search to articles published in English, using the following search string: terms (((((“Cystitis” [Mesh]) OR “Pyelonephritis” [Mesh]) OR “Prostatitis” [Mesh]) OR “Urinary Tract Infections” [Mesh]) AND “Levofloxacin” [Mesh]) AND “Ciprofloxacin” [Mesh]. We also searched the reference lists of all relevant studies included in our meta-analysis. In addition, the reference lists of all eligible studies were reviewed manually. Two investigators (CDH and SYZ) searched and evaluated studies independently. Any induced disagreement was arbitrated by a third investigator (AJZ).

### Study Selection Criteria

The study was included if met the following criteria: 1) RCT. 2) Study populations: patients with cUTIs, cystitis, pyelonephritis or bacterial prostatitis. 3) At lease one outcome for efficacy (clinical effective rate and microbial eradication rate) and safety (adverse event rate) were reported between levofloxacin and ciprofloxacin.

The following trials were excluded: non-RCT (reviews, letters, editorial comments, case reports, conference abstracts); Patients with non-UTIs; The intervention did not contain levofloxacin or ciprofloxacin, or combined with other anti-infective drugs; Outcome indicators did not include clinical effective rate, microbial eradication rate or incidence of adverse reactions; Jadad score < 3; pediatric articles, unpublished articles and non-English articles.

### Study Quality Assessment

Methodological quality of RCTs were evaluated according to Jadad scoring criteria (Jadad et al., 1996). Jadad scale was used to score the selected literature from three aspects of random allocation, including randomization, blind method and withdrawal and dropout of the study. The quality of the selected article was evaluated by two reviewers (CDH and SYZ) independently. The score of 1∼2 was classified as low quality study, and 3∼5 as high quality study.

### Statistical Analyses

Review Manager 5.3.0 (Cochrane Collaboration, Oxford, United Kingdom) was used for statistical analysis and heterogeneity testing. The heterogeneity was assessed through the chi-squared (χ^2^) test (Cochran’s Q) and inconsistency index (I^2^) (Higgins and Thompson, 2002). When χ2 *p* value >0.05 and I^2^ ≤ 50%, fixed effect model was adopted for analysis. Otherwise, the data with a I^2^ > 50% or χ^2^ *p* value ≤ 0.05 was adopted for random effect model analysis. The relative ratio (RR) was used as the pooled statistic to calculate the 95% confidence interval.

## Results

### Selected RCTs

**FIGURE 1:**
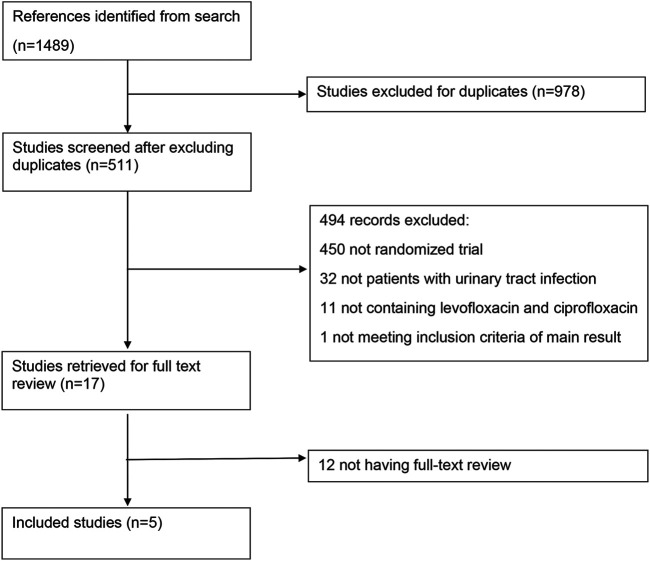
The literature screening process.

Seventeen full-text literatures were retrieved from 511 records in first screening, and 5 RCT studies were finally included (Figure 1). Table 1 showed basic characteristics of 5 studies chosen for the meta-analysis. The included literature was all of high methodological quality (2 studies with a Jadad score of 3 and 3 studies with a score of 5), among which 2 were AP (Richard et al., 1998; Klausner et al., 2007), 2 were CBP (Bundrick et al., 2003; Zhang et al., 2012), and the rest one was AP and cUTIs (Peterson et al., 2008). Details of 5 included literature could be seen in Supplementary Table S1. The research did not yield studies focusing on cystitis as a result of data absence. A total of 2,352 adult patients, all over 18 years old, were enrolled in multicenter RCTs. Levofloxacin was prescribed once a day at 250∼750 mg, orally or intravenously and patients received ciprofloxacin twice a day with a total dose of 900–1,000 mg orally or a single dose of 400 mg intravenously.

**TABLE 1:** Study characteristics of meta-analysis.

Study	Diagnosis	Sample size		Intervention		Comparator	Outcome	Jadad score
		Levofloxacin	Ciprofloxacin	Levofloxacin	Ciprofloxacin			
Bundrick et al., (2003)	CBP	136	125	500 mg qd & placebo qd, 28 d	500 mg bid, 28 d	Levofloxacin vs ciprofloxacin	①②③⑤	5
Klausner et al., (2007)	AP	146	165	Day 1–5: 750 mg qd & placebo qd Day 6–10: placebo bid	400 mg IV /500 mg orally bid, 10 d	Levofloxacin vs ciprofloxacin	①②③④⑤	5
Peterson et al., (2008)	CUTIs & AP	537	556	Day 1–5: 750 mg qd & placebo qd Day 6–10: placebo bid	400 mg/500 mg bid, 10 d	Levofloxacin vs ciprofloxacin	①②③④⑤	5
Richard et al., (1998)	AP	89	58	250 mg qd, 10 d	500 mg bid, 10 d	Levofloxacin vs ciprofloxacin	①②③④⑤	3
Zhang et al., (2012)	CBP	209	199	500 mg qd, 28 d	500 mg bid, 28 d	Levofloxacin vs ciprofloxacin	①②③④⑤	3

CBP, chronic bacterial prostatitis; AP, acute pyelonephritis; cUTIs, complicated urinary tract infections; IV, intravenously; ① = end-of-therapy clinical success rate; ② = end-of-therapy microbial eradication rate; ③ = posttherapy clinical success rate; ④ = posttherapy microbial eradication rate; ⑤ = adverse event rate;.

Treatment duration ranged from 5 to 28 days and definition for posttherapy was shown in Supplementary Table S1. We combined the 3 articles concerning AP as AP and cUTIs shared a similar dose and course of antibiotics application. As the population for analysis were various (intent-to-treat, modified intent-to-treat and microbially evaluable, respective definition could be seen in Supplementary Table S1), the pattern adopted by all corresponding articles was chosen to pool the outcome measurement except for adverse event, whose data came from all patients received 1 or more dose of studied drugs.

### Part 1. Outcomes for AP

**FIGURE 2:**
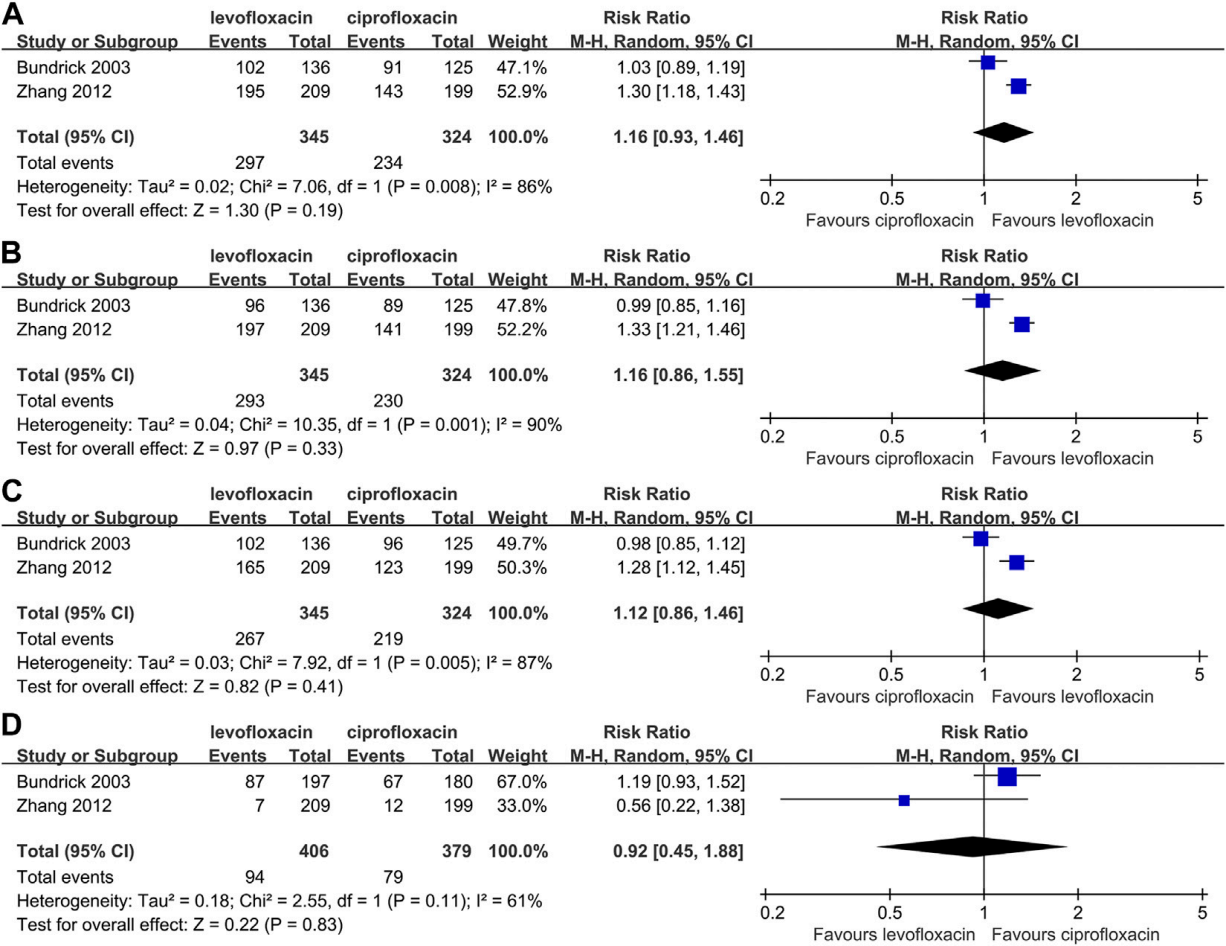
Comparison between levofloxacin and ciprofloxacin in acute pyelonephritis; 2**(A)** end-of-therapy clinical success rate; 2**(B)** posttherapy clinical success rate; 2**(C)** microbial eradication rate; 2**(D)** adverse event rate.

Two studies (Richard et al., 1998; Klausner et al., 2007) reported the rate of clinical improvement, microbial eradication and adverse event in intent-to-treat population (Figure 2). A higher incidence could be found in end-of-therapy (RR: 1.16, 95% CI: 0.93∼1.46, *p* > 0.05, Figure 2A) and posttherapy clinical effective rate (RR: 1.16, 95% CI: 0.86–1.55, *p* > 0.05, Figure 2B) for levofloxacin but without a significant divergence. For the absence of posttherapy microbial eradication rate, relevant analysis was unable to conduct. No evidence proved a significant difference with respect to microbial eradication rate at end-of-therapy (RR: 1.12, 95% CI: 0.86∼1.46, *p* > 0.05, Figure 2C) or adverse event rate (RR: 0.92, 95% CI: 0.45∼1.88, *p* > 0.05, Figure 2D). And no serious adverse event was reported.

### Part 2. Outcomes for CBP

**FIGURE 3:**
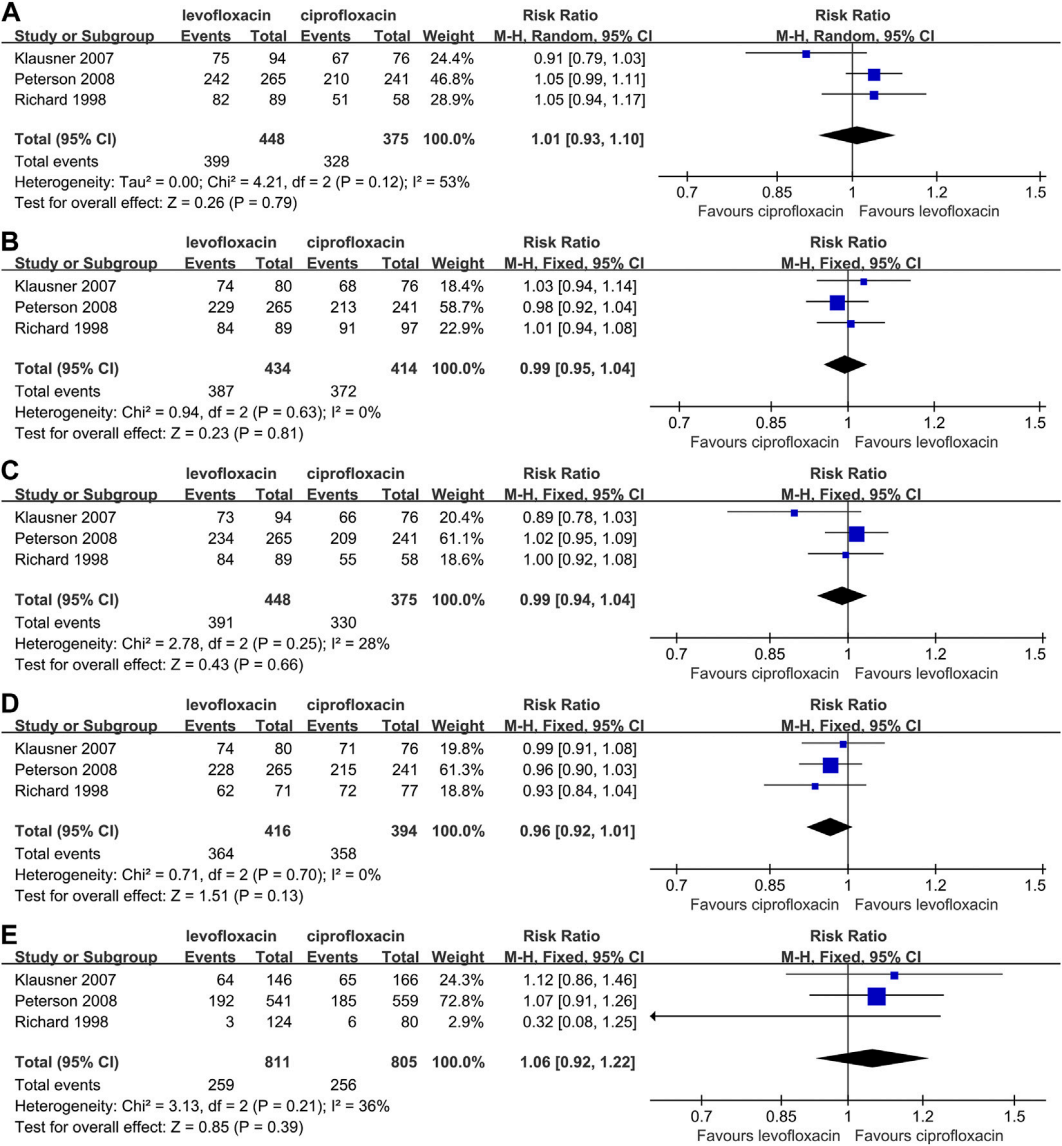
Comparison between levofloxacin and ciprofloxacin in chronic bacterial prostatitis; 3**(A)** end-of-therapy clinical success rate; 3**(B)** posttherapy clinical success rate; 3**(C)** end-of-therapy microbial eradication rate; 3**(D)** posttherapy microbial eradication rate; 3**(E)** adverse event rate.

The rate of clinical improvement, microbiological eradication and adverse event were reported by 3 articles (Bundrick et al., 2003; Peterson et al., 2008; Zhang et al., 2012) based on microbiologically evaluable population (Figure 3). As for the clinical effective rate, no matter at end-of-therapy (RR: 1.01, 95%CI: 0.93–1.10, *p* > 0.05, Figure 3A) or posttherapy (RR: 0.99, 95%CI: 0.95–1.04, *p* > 0.05, Figure 3B), there was no significant difference between the 2 drugs. What’s more, the statistical difference of end-of-therapy (RR: 0.99, 95% CI: 0.94–1.04, *p* > 0.05, Figure 3C) or posttherapy (RR: 0.96, 95% CI: 0.92–1.01, *p* > 0.05, Figure 3D) microbial eradication rate was unsignificant.

When compared directly, the levofloxacin and ciprofloxacin did not show a significant divergence in adverse event rate (RR: 1.06, 95% CI: 0.92∼1.22, *p* > 0.05, Figure 3E). 5/146 severe adverse event in levofloxacin subject, with one case of urosepsis, and 6/166 in ciprofloxacin were noted (Klausner et al., 2007). The corresponding number is 17/537, 15/556 in another study (Richard et al., 1998) and one was considered related to allergic reaction to levofloxacin. No treatment-related death case was mentioned by all 3 studies.

## Discussion

UTIs are becoming a global health issue and induce significant quantity of societal cost (Flores-Mireles et al., 2015). Timely and effective antibiotic treatment, together with nutraceutical, could reduce the long-term damage to reproductive system (Mongioi et al., 2016). In the treatment of UTIs, although the guidelines list levofloxacin and ciprofloxacin as first-line drugs for cUTIs and AP, no evidence-based research has proven the comparative advantage of the either one. Currently, there is no meta-analysis on this issue. Under these conditions, we performed a latest systematic review and pooled analysis of all available trials. All involved trials were RCTs with high level of evidence (mean Jadad score = 3.9), which gave our study enough authority on this topic.

For AP and CBP treatment, the analysis did not show significant statistical difference in terms of end-of-therapy or posttherapy clinical effective rate and microbial eradication rate. The adverse event rate shared a similar finding. As for the treatment of CBP, however, Zhang (Zhang et al., 2012) reported that levofloxacin was with higher efficacy, lower disease reoccurrence and adverse event rate in Chinese patients. The isolated bacteria from urine sample could account for this controversy. The most common uropathogen was *E. coli* in this trial while *Enterococcus faecalis* ranked first and *E. coli* got a fifth place in a similar American study (Bundrick et al., 2003), which did not find the difference between the two drugs. Nowadays, the prevalence of resistant *E. coli* rose in community-acquired urinary tract infection (Lee et al., 2018). Based on the fact that the minimal inhibitory concentration of levofloxacin for resistant *E. coli* isolates were lower than that of ciprofloxacin (Becnel Boyd et al., 2009), hypothesis could be raised naturally that bacterial spectrum and corresponding sensitive antibiotics for Chinese patients were not totally consistent with other countries and therefore the dispute arised. But it did not mean that levofloxacin was recommended for *E. coli*-induced UTIs treatment until other first-line drugs failed to work, based on the fact that gram-negative bacteria was least susceptible to levofloxacin and ciprofloxacin in the sensitivity test both in China and United States (Lu et al., 2012; Bouchillon et al., 2013).

Limited by existing studies, the hypothesis could be hardly confirmed unless RCTs with a large sample performed. But it was consistent with pharmacologic research. Fluoroquinolones exhibit concentration dependent killing (Craig, 1998) and levofloxacin was characterized by a nearly twice renal excretion rate (84%) than ciprofloxacin (43%), possessed with higher urinary bactericidal titers and long lasting time (Naber, 2001). Drusano et al. showed that the prostate/plasma ratio of more than 70% of the subjects exceeded 1.0, indicating that levofloxacin was able to penetrate the prostate and suitable for local infection (Drusano et al., 2000). Wagenlehner et al. reported that the blood concentration of levofloxacin in healthy volunteers was higher than that of ciprofloxacin at a single dose (Wagenlehner et al., 2006). It proved reasonable explanation for the non-inferiority of levofloxacin, whose minimum inhibitory concentration for uropathogen was higher in disk diffusion, compared with ciprofloxacin.

For the adverse event, the 2 drugs were comparable and no noteworthy serious or death case came into publication. The most common side effect were digest tract symptom (flatulence and diarrhea) and central nervous symptom (headache, dizziness and nausea), which was consistent with existing report (Stahlmann and Lode, 2013). No adverse event was considered directly related to treatment except for an allergic reaction case (Peterson et al., 2008). Known severe side effect, such as QT prolongation, seizure and tendon rupture (Stahlmann and Lode, 2013), were not reported by all 5 trials. All mentioned above proved that levofloxacin and ciprofloxacin were both with safety in clinical application.

There were several limitations in our meta-analysis. First, the quantity of available studies were small, resulting in inadequate statistical confidence. RCTs with larger scale were necessary to furtherly explore the answer. Second, the difference in standard course and dose of the 2 drugs could bias the result. Some researchers held the view that as part of the short-course therapy, the course of levofloxacin treatment concluded 5 days sooner than that of ciprofloxacin (5 vs. 10 days), potentially biasing the efficacy assessments in favor of ciprofloxacin (Klausner et al., 2007; Peterson et al., 2008). Third, it was the study design and the inclusion criteria of the individual RCTs that may be responsible for failing to reveal the differences between levofloxacin and ciprofloxacin. Actually, most of these RCTs included were to show noninferiority between agents for drug registration and approval purposes. Therefore, they may fail to show clinical superiority of any antibiotic over another. We have good reasons, though, to believe that the high quality of included RCTs could make up for this shortcoming.

## Conclusion

At present, this is the first evidence-based research comparing efficacy and safety between levofloxacin and ciprofloxacin as for urinary tract infection. There is no significant difference between the 2 drugs in end-of-therapy or posttherapy clinical success rate, microbial eradication rate or adverse event rate.

## Data Availability

The original contributions presented in the study are included in the article/Supplementary Material, further inquiries can be directed to the corresponding authors.
