# Supplementary material for: Levofloxacin Versus Ciprofloxacin in the Treatment of Urinary Tract Infections: Evidence-Based Analysis
Source: Front Pharmacol. 2021 Apr 8;12:658095. doi: 10.3389/fphar.2021.658095 (PMC8060646; doi:10.3389/fphar.2021.658095)
Supplement: Supplementary file 1 [file table1.docx]

Supplementary table 1. Detailed information of the 5 studies included in the meta-analysis

| Study | Patients enrolled | Gender(f/m) | | Age  (Mean ± SD) | | Population | Definition for posttherapy |  |
| --- | --- | --- | --- | --- | --- | --- | --- | --- |
|  |  | Levofloxacin | Ciprofloxacin | Levofloxacin | Ciprofloxacin |  |  | |
| Bundrick 2003 | 383 | 0/383 | | Not reported | | ITT | 5-18 days after therapy |  |
| Klausner 2007 | 311 | 138/8 | 161/4 | 38.9 ± 17.96 | 39.4 ± 17.05 | mITT & ME | 10 to 14 days postlevofloxacin  5 to 9 days postciprofloxacin | |
| Peterson 2008 | 1109 | 330/207 | 336/220 | 54.2 ± 20.0 | | mITT & ME | 10 to 14 days postlevofloxacin  5 to 9 days postciprofloxacin |  |
| Richard 1998 | 147 | 80/9 | 49/9 | 41 ± 20.8 | 34 ± 17.5 | ME | 5-9 days after therapy | |
| Zhang 2012 | 471 | 0/471 | | 33.4 ± 8.1 | 33.5 ± 8.5 | ITT | 6 months after therapy | |

ITT: intent-to-treat; mITT: modified ITT, referred to all ITT subjects who had a clinical diagnosis of AP and who had a positive urine culture (≥ 10^5^ cfu/mL) and ≤ 2 uropathogens at study entry. ME: microbiologically evaluable, referred to mITT subjects who additionally met all other major evaluability criteria like no follow-up lost and no dosing deviations.
